# Phase Structure Evolution of the Fe-Al Arc-Sprayed Coating Stimulated by Annealing

**DOI:** 10.3390/ma14123210

**Published:** 2021-06-10

**Authors:** Tomasz Chmielewski, Marcin Chmielewski, Anna Piątkowska, Agnieszka Grabias, Beata Skowrońska, Piotr Siwek

**Affiliations:** 1 Institute of Manufacturing Technologies, Warsaw University of Technology, Narbutta Str. 85, 02-524 Warsaw, Poland; beata.skowronska@pw.edu.pl (B.S.); siwek_piotr@wp.pl (P.S.); 2 Łukasiewicz Research Network—Institute of Microelectronics and Photonics, Al. Lotników 32/46, 02-668 Warsaw, Poland; Marcin.Chmielewski@imif.lukasiewicz.gov.pl (M.C.); Anna.Piatkowska@imif.lukasiewicz.gov.pl (A.P.); Agnieszka.Grabias@imif.lukasiewicz.gov.pl (A.G.)

**Keywords:** Fe-Al type intermetallics, phase synthesis, arc sprayed coatings

## Abstract

The article presents the results of research on the structural evolution of the composite Fe-Al-based coating deposited by arc spray with initial low participation of in situ intermetallic phases. The arc spraying process was carried out by simultaneously melting two different electrode wires, aluminum and low alloy steel (98.6 wt.% of Fe). The aim of the research was to reach protective coatings with a composite structure consisting of a significant participation of Fe_x_Al_y_ as intermetallic phases reinforcement. Initially, synthesis of intermetallic phases took place in situ during the spraying process. In the next step, participation of Fe_x_Al_y_ fraction was increased through the annealing process, with three temperature values, 700 °C, 800 °C, and 900 °C. Phase structure evolution of the Fe-Al arc-sprayed coating, stimulated by annealing, has been described by means of SEM images taken with a QBSD backscattered electron detector and by XRD and conversion electron Mössbauer spectroscopy (CEMS) investigations. Microhardness distribution of the investigated annealed coatings has been presented.

## 1. Introduction

Transition iron aluminides are attractive coating materials with specific properties, especially in comparison with nickel or chromium-based materials. Fe-Al intermetallic systems have been among the most intensively studied over the last few decades [1]. Many intermetallic applications of the Fe-Al system relate to protective coatings made by different methods, like laser cladding [2,3], D-gun spray [4,5,6], flame spray [7,8], arc spray [9,10,11], cold gas spraying [12], and plasma transferred arc cladding [13], some using the strategy of employing the elemental powder materials [14]. Intermetallic phases, due to their advantages, are increasingly often used as a surface material, whose purpose is to work at high temperatures [15,16]. They are significantly resistant to oxidation, carburizing, and sulfation at high temperatures (up to 900 °C) [17,18]. Additionally, they are highly resistant to erosion [19] and cavitation [20,21], and have relatively low density and low prices compared to corrosion-resistant [22,23] and acid-resistant steel [24], which require application expensive elements, such as Cr, Ni, Mo [25,26,27]. The intermetallics owe their special properties to their ordered structures with strong chemical bonds and simultaneous dense packing of atoms in crystal lattices, which leads to reduced diffusion velocity, creep resistance, and resistance to high-temperature corrosion [28,29,30,31].

There are reports in the literature that have described the processes of in situ manufacturing of intermetallic phases on a surface layer by the alloying of components [32,33]. Numerous cases of annealing-stimulated intermetallic synthesis are known in the literature [34,35,36,37], which could also be used as a part of the joining procedure [22]. The main disadvantage that limits the use of Fe-Al intermetallics is their brittleness at room temperature and the difficulty of shaping ready-made elements to desired dimensions by means of mechanical machining methods [38,39]. Due to the significant differences in properties like melting point and specific heat of Fe and Al, it is difficult to obtain materials with a reproducible composition and homogeneous structure [40].

The method of producing a composite material using the Fe-Al intermetallic phase proposed in the article is a continuation of the procedure proposed in [10] and may constitute an alternative to the currently used solutions, which are usually much more expensive and based mainly on ready-to-use intermetallic powders [41,42].

## 2. Materials and Experimental Procedure

The initial investigation [10] and its results showed that a 0.5 mm thick and dense Fe-Al composite coating with uniformly distributed Fe and Al particles can be deposited by arc spraying onto the substrate of an S355 JR steel plate (50 × 100 × 5 mm^3^). The coating obtained included two main metallic phases based on the Fe (bcc) and Al (fcc) structure. However, Fe-Al precipitations have been revealed on the boundary grains as well as on the Al and Fe matrix. Volume fraction of intermetallic phases in the sprayed coating was too low to use XRD method to investigate them. Results showed that both SEM and EDS analyses confronted with Mössbauer spectroscopy analysis confirmed presence of FeAl intermetallic phases in the structure with varying atomic factors of iron and aluminum, including approximately 50-50% and 80-20% at the structure of the coating. Volume fraction of Fe_x_Al_y_ phases was between 7% and 10%.

In this paper, an iron/aluminum composite coating deposited by arc spraying using iron and aluminum wires in the condition described in [10] was annealed at different temperature (700 °C, 800 °C, and 900 °C) values for 2 h to aim at increasing the volume fraction of iron aluminide intermetallic in composite coating. The parameters of the thermal annealing cycle were selected on the basis of a literature analysis [43,44,45]. Annealing processes have been realized in vacuumed atmospheres (5 × 10^−5^ Tr) with an experimental vacuum chamber with induction heating, designed and built in the welding department of Warsaw University of Technology (Poland). After annealing, the samples were cooled and prepared for metallographic examination in the cross-section and on the surface. The specimens were grounded in the cross-section and on the coating’s surface with abrasive paper up to 2000 grits, polished to obtain a mirror-finished surface for microstructure observation and chemical and phase composition analysis. The composite, multi-phase Fe-Al arc-sprayed coating on the steel substrate after annealing was analyzed via scanning electron microscope Auriga produced by the Zeiss company (Oberkochen, Germany). The microstructure and phase structure of the coating after annealing were investigated by using SEM, XRD, and Mössbauer spectroscopy analogously to the study its structure before annealing for comparison. X-ray diffraction measurements were performed using the Siemens D500 powder diffractometer (Siemens, Munich, Germany), equipped with a high-resolution semiconductor Si:Li detector and Kα1,2Cu radiation λ = 1.5418 Ǻ. Conversion electron Mössbauer spectroscopy (CEMS) has been carried out using a constant acceleration home-made Mössbauer spectrometer (designed and built by Łukasiewicz Research Network—Institute of Microelectronics and Photonics) with integrated gas flow electron counter. Measurements were performed at room temperature with the use of a ^57^Co-in-Rh source. The annealed coatings were probed up to about 200 nm in depth. As a supplement to the assessment of structural changes, the distribution of the coating’s hardness in the cross-section along four parallel lines up to the surface layer of substrate material were performed according to the Vickers method using the Leitz–Wetzlar microhardness tester (LEICA, Wetzlar, Germany) (load 100 g for 10 s).

## 3. Results and Discussion

### 3.1. SEM Investigation of Annealed Coatings

**Figure 1:**
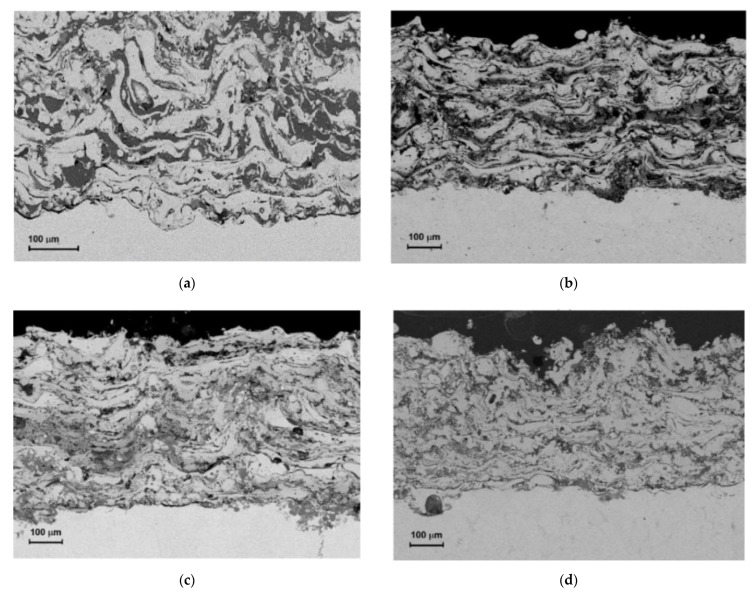
SEM/QBSE images of as-arc-sprayed Fe-Al-type coating (**a**), and after annealing for 2 h, at respectively: (**b**) 700 °C, sample 1; (**c**) 800 °C, sample 2; (**d**) 900 °C, sample 3.

SEM images taken throughout the entire coating (Figure 1) revealed a large variety of phases and their generally lamellar distribution nature, specific to thermally sprayed coatings [46,47]. The aluminum wire is chemically active in the arc spraying process, such that melted particles are already oxidized in metallization stream and the obtained coatings always contain oxide films, mainly present in the areas between of lamellar grains of higher aluminum content.

As a result of high kinetic energy, temperature, and the speed of melted particles in the arc-spraying process, the Fe-Al-type coatings that were about 500 μm thick showed a lamellar microstructure with an inhomogeneous phases distribution where a few spherical non-molten particles were visible (Figure 1a). Based on SEM/QBSE images, different shades of gray were observed for indicated phases with different atomic masses in the backscattered electron (QBSE) detector. 

Various shades of gray areas in the SEM/QBSE images correspond to different phases—predominant Fe(Al) solid solution with high iron is indicated by the lightest grains and the aluminum-rich phases, including oxides and spinels, are represented by the darkest grains in Figure 1a.

The local occurrences of disordered and dispersed intermetallic Fe-Al-type phases in various Al content must be also emphasized (presented as the varied degree of grayness in individual grains in the QBSE examination and slightly less bright than these high-Al grains—Figure 1a).

The high degree of chemical inconsistency of the arc-sprayed Fe-Al-type coating also proves its intermetallic phase inhomogeneity, especially when one takes into consideration the fact that a big range of acceptable changes in the alloying elements contents in the Fe-Al intermetallic phase solutions [46,47]. The comparative analysis in the cross-sections of the Fe-Al type coating (Figure 1) indicate that the Fe-Al arc-sprayed coating (Figure 1a) is not uniform in the phase structure and morphology throughout its thickness [10]. However phase structure of the coating has been significantly changed, in the direction of higher homogeneity, after annealing sample 1 at 700 °C (Figure 1b), sample 2 at 800 °C (Figure 1c), and sample 3 at 900 °C (Figure 1d).

As the Fe-Al type arc-sprayed coatings were annealing at high temperatures, the thermal and hardness stability of the Fe-Al type intermetallic coating (such as the change in the morphology and chemical composition of the lamellar structure, phase change susceptibility, and the degree of strengthening of the coating) was analyzed after heating at a high-temperature (respectively 700 °C, 800 °C, and 900 °C) for 2 h.

### 3.2. EDX Measurements

#### 3.2.1. Arc-Sprayed Fe-Al-Type Coating Annealed at 700 °C 

The basis for identification of the structural analysis and inhomogeneity of the chemical composition (phase composition) in the arc-sprayed Fe-Al type coatings after they were annealed at high temperatures were the EDX results of point and linear microanalysis of the chemical composition, as well as mapping of Fe, Al, and O elements. 

**Figure 2:**
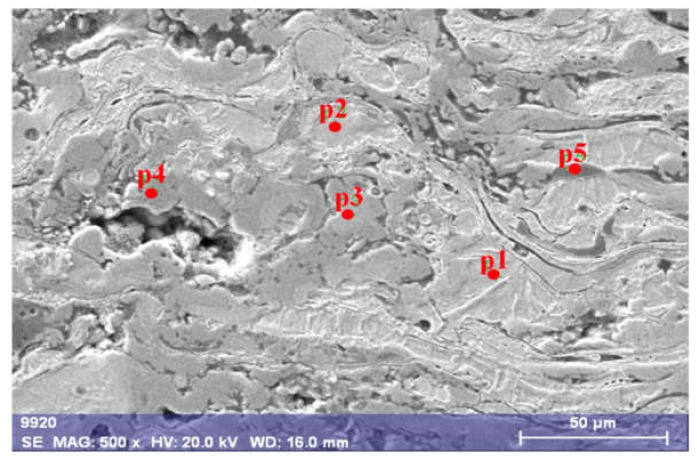
Microstructure of arc-sprayed Fe-Al-type intermetallic coating after annealing at 700 °C for 2 h with hypothetical phase identification based on results of EDS point analysis (in Table 1).

Based on the results of SEM/EDX point microanalysis, it was shown (Figure 2) that the layered arrangement of grains of the arc Fe-Al-type coating annealed at 700 °C revealed a considerably varied chemical composition with an extended range of solid solution from about 6 to 37 at.% Al (Table 1). This wide range of compositions implies the occurrence of grains based on the low-aluminum Fe(Al) solid solution grains observed as the bright gray areas in the BSE image (p1 and p2 in Figure 2).

**Table 1:** Chemical composition of as-arc-sprayed Fe-Al-type coating after annealing at 700 °C/2 h based EDX point microanalysis according to Figure 2.

Designation of Grain Area According to Figure 2	Content, at. %							
	C	O	Al	Si	Mn	Fe	Au	Occurrence
p1	~9	~1	6.47	0.48	0.66	81.49	0.15	often
p2	~10	-	7.69	0.84	0.60	80.62	0.13	often like p1
p3	~12	~19	24.90	0.55	0.72	41.85	0.10	rare
p4	~4	~51	6.33	0.29	0.38	36.25	0.09	rare like p3
p5	~15	~35	35.76	0.14	0.64	12.87	0.09	very rare

Annealing at 700 °C for 2 h also caused a local presence of disordering secondary solution based on Fe-Al phases ranging from 25 to 36 at.% Al and a significant content of oxygen (p3 and p5 in Figure 2). However, near some Fe-Al phases, depletion of Al entailed a local occurrence of oxides in the form of spinels (p4 in Figure 2), as well as aluminum oxides created between lamellar grains (p5 in Figure 2) where Fe was doped from the matrix of Fe-Al intermetallic material.

In the discussion of the EDX analysis, the identified trace elements were not taken into account (Table 1), as the contaminations of Si, Mn, and Au could be caused by polishing of the samples as well as sputtering with a thin Au layer about 10 nm thick in order to avoid issues with the electrical charge of the primary electron beam in the preparation of the cross-sections of the samples that were obtained using a non-conductive resin as a matrix.

The relative content of these contaminations was below 1 at.% and can be considered as negligible. In the case of light elements such as oxygen and carbon, the analysis was semi-quantitative. The significant presence of carbon was most probably related to the preparation of cross-sections of the samples, where during the polishing process the resin could be transferred to the studied surface of the sample.

**Figure 3:**
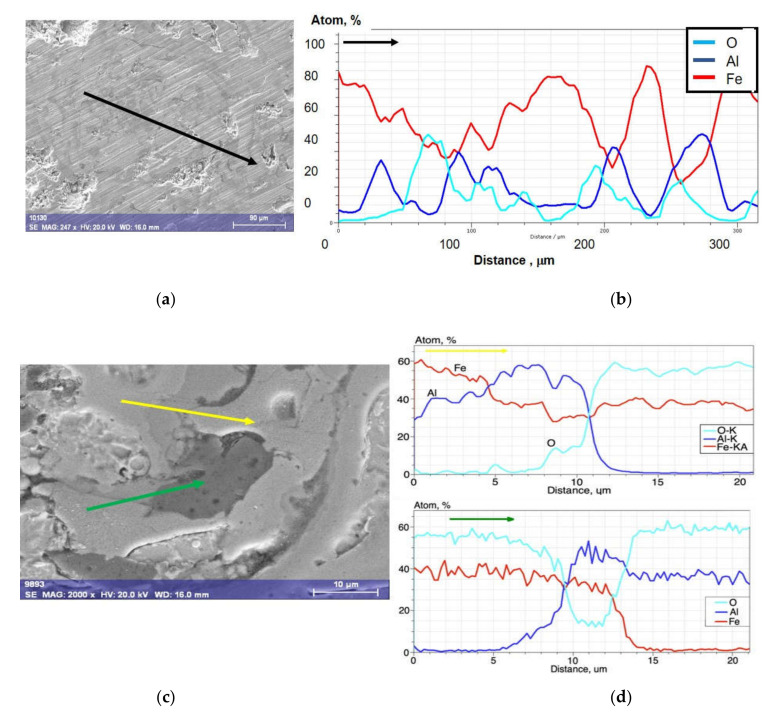
Linear chemical analysis of as-arc-sprayed Fe-Al-type coating after annealing at 700 °C/2 h: (**a**) SE image of the surface of the coating, (**b**) linear distribution of elements along the black arrow marked on Figure 3a, (**c**) SE image with details of the layer cross-section, (**d**) linear distribution of elements along the black narrow marked on Figure 3a, (**c**) SEM image with details of the layer cross-section, (**d**) linear distribution of elements along the yellow and green arrows marked on Figure 3c.

**Figure 4:**
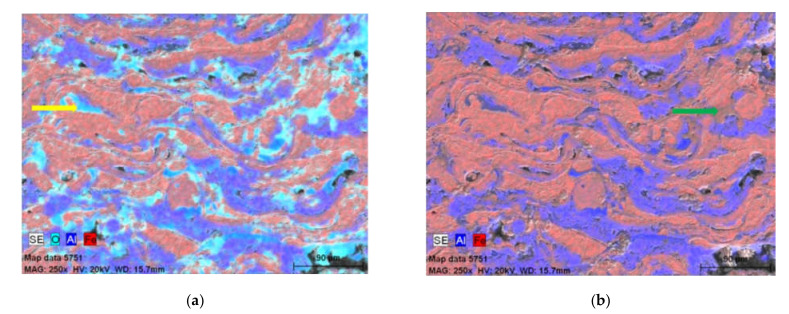
EDX maps of elemental distribution in cross-section of the Fe-Al-type coating annealed at 700 °C: (**a**) O, Al, Fe; (**b**) Al, Fe.

The basis for identification of oxides in the Fe-Al-type coating after annealing at 700 °C were the results of a linear microanalysis of the chemical composition (Figure 3), as well as mapping of Fe, Al, and O elements (Figure 4), which proved that oxygen, apart from oxide spinels, is mainly present in the areas of grains of higher aluminum content (imaged as dark grey and grey), but also in the light grey areas identified as disordering secondary solid solution based on Fe-Al phases with decreased Al content.

Additionally, the results of the linear EDS measurements (Figure 3a,b) performed on the representative surface of the Fe-Al-type coating annealed at 700 °C showed very different proportions of the Al-Fe elemental ranging from 10 to 50 at.% Al along the line with a length of approx. 20 µm. The linear EDX results of changes in the proportions and chemical compositions at the grain boundary cross-sections of the coatings are shown in Figure 3c,d. In the region marked with the yellow arrow, the phase composition of roughly 50 at.% Fe was observed up to about first 10 µm. The measurement along the green arrow indicates at one end Fe oxide, whereas on the other end there is Al oxide. In the middle, a mixed Fe-Al-O phase is observed.

This is a confirmation of the fact that the lamellar grains created in the arc-spraying conditions after annealing at 700 °C exhibited certain composite features due to the different Fe-Al type phases as components of the structure inherited from the arc-spraying wire material, only with inconsiderable evidence of the phase transformation during heating at 700 °C for 2 h. The aluminum wire is chemically active in the arc-spray process, such that all melted particles are already oxidized in the obtained coatings and always contain oxide films inside the coating and at the internal interfaces. It is mainly formation of the oxide films (the oxidized blue areas of the coating structure on EDX maps, Figure 4a) that brings phase transformation during heating at 700 °C for 2 h.

#### 3.2.2. Arc-Sprayed Fe-Al-Type Coating Annealed at 800 °C

**Figure 5:**
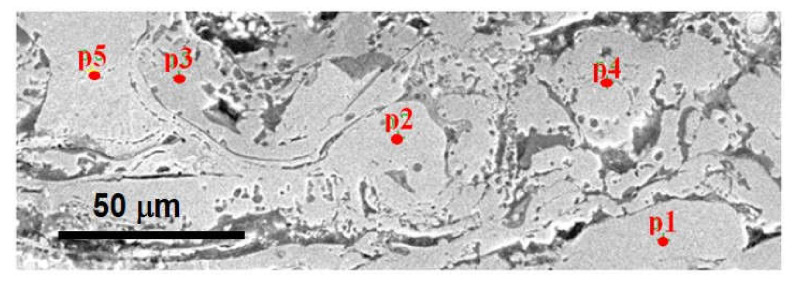
Microstructure of arc-sprayed Fe-Al-type intermetallic coating after annealing at 800 °C for 2 h with hypothetical phase identification based on results of EDX point analysis (in Table 2).

**Figure 6:**
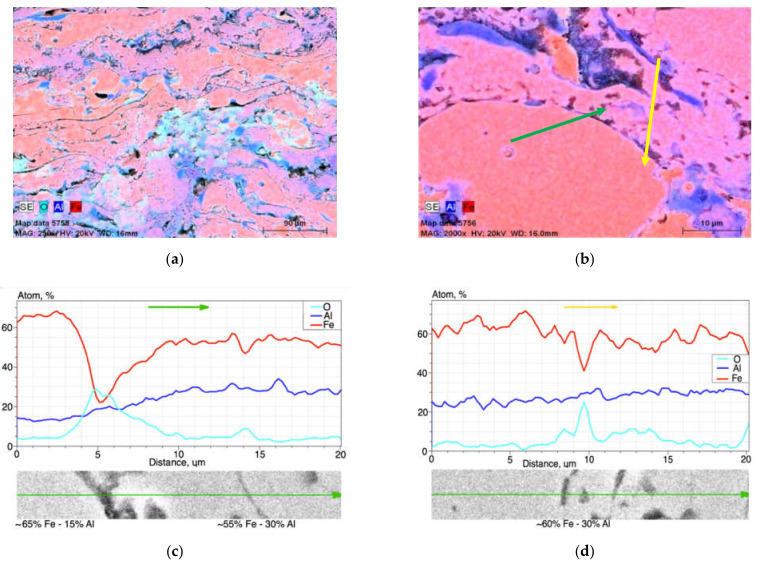
EDX results of elements distribution in cross-section of the coating annealed at 800 °C: maps of (**a**) O, Al, and Fe and (**b**) Al and Fe, as well as plots of the linear distribution of elements along two lines, respectively—(**c**) green and (**d**) yellow—as marked on (**b**).

After heat treatment at 800 °C for 2 h, the SEM/EDX results (Figure 5 and Figure 6) revealed the inhomogeneous lamellar structure characteristic-like arc spraying with a varied chemical composition based on the Fe-Al-type phases and oxides, identified mainly in the interlamellar grain boundaries of the coating (Figure 5 and Figure 6). Based on the results of scanning electron microscopy and point EDX microanalysis and mapping, it was shown (Figure 5 and Figure 6a,b) that the grains based on the Fe-Al phases had the range of secondary solid solution from ~14 to ~33 at.% Al (Table 2).

**Table 2:** Chemical composition of as-arc-sprayed Fe-Al-type coating after annealing at 800 °C/2 h based on EDX point microanalysis according to Figure 5.

Designation of Grain Area According to Figure 5.	Content, at.%							
	C	O	Al	Si	Mn	Fe	Au	Occurrence
p1	~9	-	21.60	0.92	0.57	67.49	0.08	often
p2	~8	~3	17.34	0.70	0.39	69.55	0.07	often
p3	~9	-	32.86	0.40	0.32	56.63	0.21	rare
p4	~9	-	25.84	0.74	0.53	63.18	0.09	often
p5	~13	~8	13.93	0.63	0.43	62.99	0.26	medium

The share of the dark phase related to Al and the Fe-Al intermediate phases were increased. Areas in the pink color in Figure 6a,b were suitable for Fe-Al phases. The more violet the color of the surface in the structure, the greater the proportion of aluminum. The SEM/EDX results shown in Figure 5 and Figure 6 and Table 2 allowed identification of the fact that in the Fe-Al-type coating annealed at 800 °C/2 h, the most common phase was low-aluminum Fe_3_Al at.%, as shown by the disordered secondary solid solution observed in the brightest areas of the SEM/BSE image (Figure 5) and in the pink Fe- and Al-mapping (Figure 6a,b). In the plots of the variations of the aluminum, oxygen, and iron in the linear EDX analyses (Figure 6c,d), the peak oxygen oscillations corresponded to the passage of the analyzing beam of electrons through the oxides, which was the inherent structural component of the Fe-Al-type coating annealed at 800 °C/2 h.

#### 3.2.3. Arc-Sprayed Fe-Al-Type Sample 3 Annealed at 900 °C 

**Figure 7:**
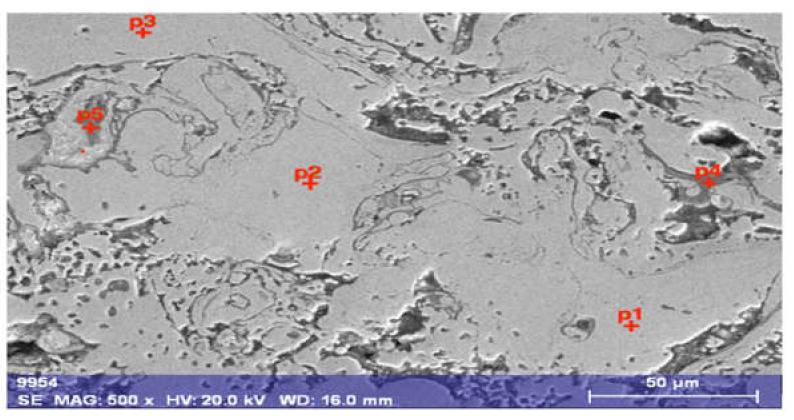
Microstructure of arc-sprayed Fe-Al-type intermetallic coating after annealing at 900 °C for 2 h with hypothetical phase identification based on results of EDS point analysis (in Table 3).

**Figure 8:**
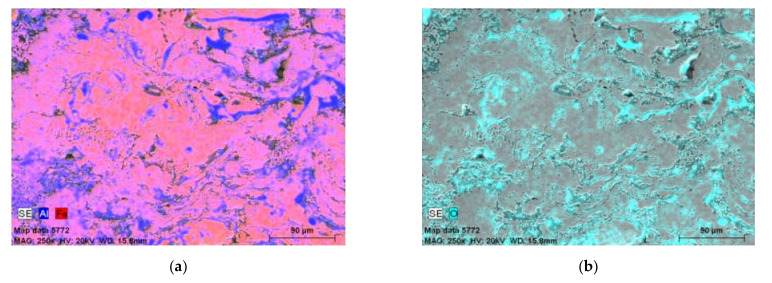

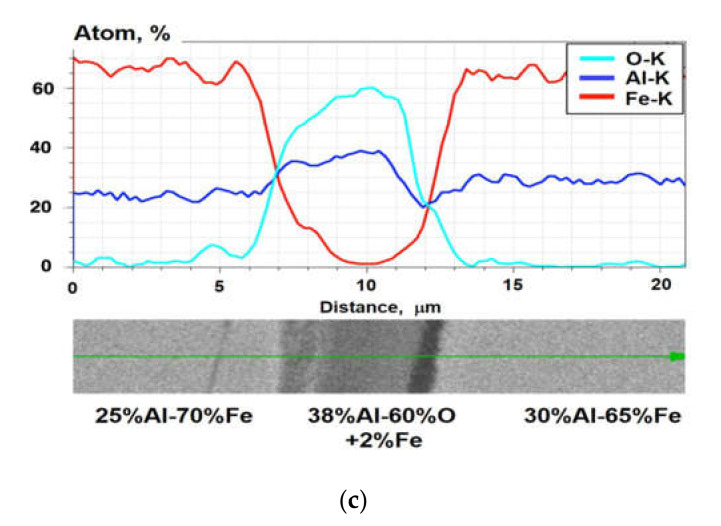
EDX results of elemental distribution in cross-section of the coating annealed in 900 °C: maps of (**a**) Al and Fe and (**b**) O, as well as (**c**) plots of the linear distribution of elements along green line.

The morphology of arc-sprayed Fe-Al-type coating annealed at 900 °C for 2 h was related to the presence of dispersed intermetallic phases in the various stages of ordering (depending on the Al content), as shown in Figure 7 and Figure 8, where the different phases on the cross-section of the coating were revealed.

**Table 3:** Chemical composition of as-arc-sprayed Fe-Al-type coating after annealing at 900 °C/2 h based EDX point microanalysis according to Figure 7.

Designation of Grain Area According to Figure 7	Content, at.%							
	C	O	Al	Si	Mn	Fe	Au	Occurrence
p1	~15	-	21.86	0.55	0.64	61.78	0.10	often
p2	~9	-	21.64	0.79	0.66	67.67	0.15	often like p1
p3	~8	-	23.57	0.48	0.63	67.11	0.14	often like p1
p4	~6	~56	34.62	0.21	0.94	1.22	0.05	rare -precipitation
p5	~4	~55	34.47	0.04	0.23	4.47	0.08	rare

After heat treatment, the microstructure lost its lamellar-like structure, which appeared to have more homogeneity at this point but was still not quite regular. In the middle area of the EDX map in Figure 8a, the phase indicated by the red color was rich in Fe and, it coexisted with the dominant Fe-Al phase in bright weight contrast marked in pink in the EDX Fe and Al maps. On the other hand, the Al-rich phase occurred sporadically, mainly crystallized as narrow lamellas between the matrix areas, and it was also highly oxidized, as presented on Figure 8c.

### 3.3. XRD Analysis

In order to analyze the phase structure of the coatings, the XRD analysis was performed for three representative samples annealed at the temperatures of 700 °C, 800 °C, and 900 °C.

**Figure 9:**
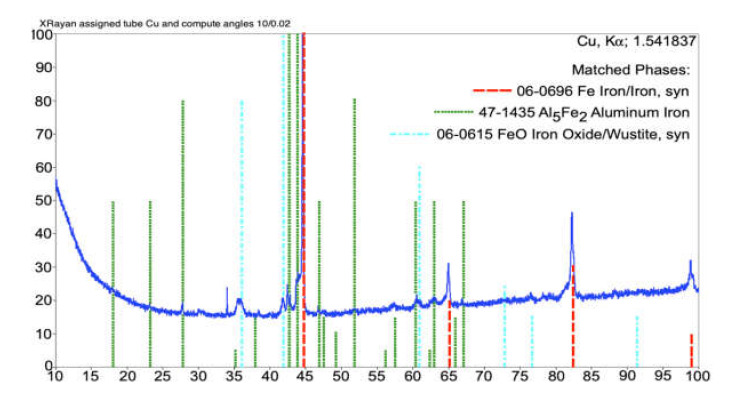
XRD diffraction pattern of the arc-sprayed Fe-Al coating after 700 °C annealing.

Figure 9 presents a diffraction pattern that implies that the phase structure of the studied coating after annealing at 700 °C consisted mainly of the metallic phase based on the bcc Fe phase, which was accompanied by the intermetallic Al_5_Fe_2_ phase. The dominant bcc phase was most probably an Fe(Al) solid solution with a substantial content of the solvent, which was suggested by a significantly larger value of the lattice constant as compared to pure Fe. A small fraction of an iron oxide phase with the wustite-like structure (FeO) was also seen in the XRD pattern. The volume fraction of this phase in the structure was approximately 5%; however, after annealing at 800 and 900 °C, it dropped below the level of XRD detection.

**Figure 10:**
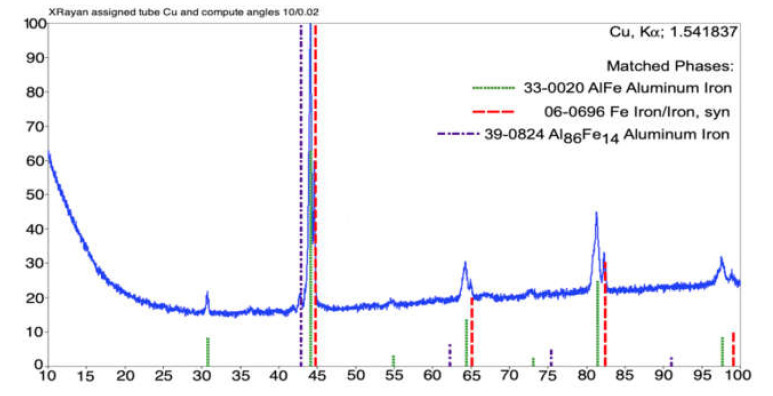
XRD diffraction pattern of the arc-sprayed Fe-Al coating after 800 °C annealing.

**Figure 11:**
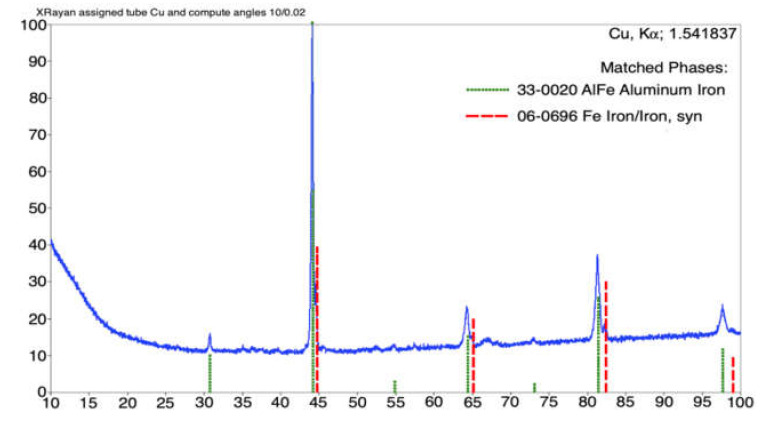
XRD diffraction pattern of the arc-sprayed Fe-Al coating after 900 °C annealing.

The XRD pattern obtained for the coating annealed at 800 °C is shown in Figure 10. The phase structure of the analyzed coating consisted of a substantial fraction of the FeAl intermetallic phase, as well as the bcc Fe-based metallic phase and a metastable Al86Fe14 phase. Figure 11 presents the diffraction pattern of the sample annealed at 900 °C, which revealed two main phases. The dominant intermetallic FeAl phase coexisted with the bcc Fe-based metallic phase.

The XRD studies were complemented by conversion electron 57Fe Mössbauer spectroscopy measurements, which provided additional information regarding the atomic environment of iron atoms in the studied coatings.

### 3.4. Mössbauer Spectroscopy Results of Annealed Coatings

**Figure 12:**
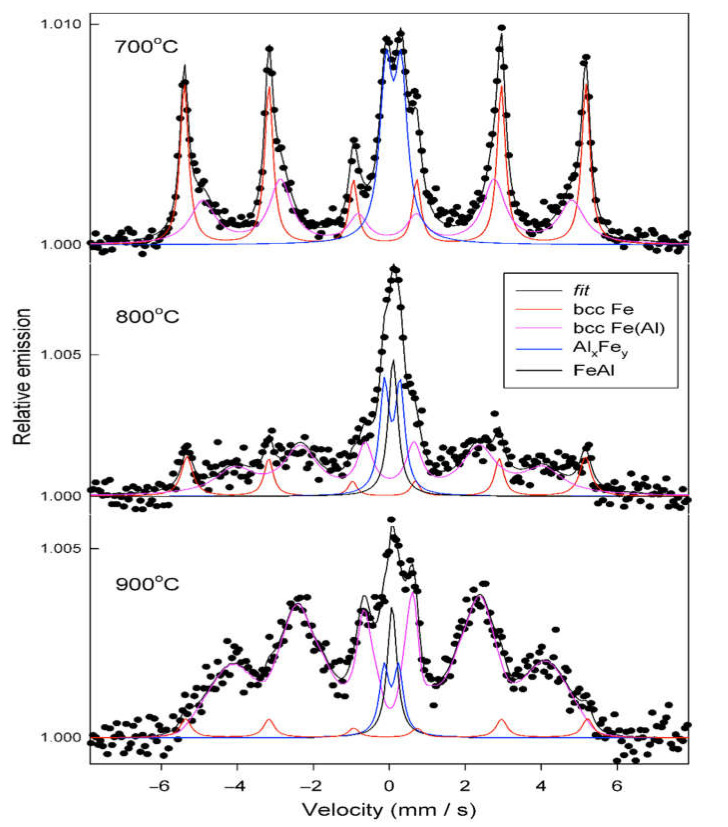
Conversion electron Mössbauer spectra of the coatings annealed at 700–900 °C.

The identification of iron-containing phases in the samples was done on the basis of hyperfine parameters, such as the hyperfine field, isomer shift, and quadrupole splitting, which were determined for the particular spectral components. Isomer shift values are related to the α-Fe standard. The conversion electron Mössbauer spectra measured as a function of annealing temperature of the coating are shown in Figure 12. The CEMS spectra revealed a distribution of Fe atoms in two magnetic and two paramagnetic Fe-Al environments in the annealed coatings.

The qualitative and quantitative analyses of the phase composition were performed based on fitting of the spectra with the use of the following spectral components:

- A magnetically split component (sextet) with the hyperfine field of 32.7 T, assigned to bcc Fe atomic environments without Al atoms as the nearest neighbors; however, some Al atoms were present in the remote vicinity of Fe atoms, thus causing a reduction of the hyperfine field of 32.95 T, characteristic for the pure bcc Fe phase [10,48,49];
- A sextet with broad lines and average hyperfine field values in the range of 25−30 T, originating from bcc Fe(Al) disordered solid solution;
- A quadrupole doublet with the quadrupole splitting of 0.40−0.44 mm/s and the isomer shift ranging from 0.19 to 0.23 mm/s, assigned to a paramagnetic Al-rich Al_x_Fe_y_ phase;
- A single line with the isomer shift of 0.22 mm/s, assigned to a paramagnetic intermetallic bcc FeAl phase.

The CEMS spectrum obtained after annealing at 700 °C was qualitatively similar to the spectrum of the untreated sample studied previously [10]. It consisted of the same three spectral components (1)–(3); however, the contribution of the quadrupole doublet (3) related to the formation of the Al-rich Al_x_Fe_y_ phase was markedly larger than for the unannealed sample. Annealing at 800–900 °C induced structural changes in the Fe-Al coating, which were clearly seen in the CEMS spectra as the intensity of the bcc Fe spectral component (1), dominating at 700 °C, which decreased significantly at higher temperatures in favor of the components related to binary Fe-Al phases. The broad sextet (2) revealed a distribution of the hyperfine field, particularly observed for the samples annealed at 800–900 °C. The hyperfine field values covered a wide range from about 10 T up to 33 T. The average values of the hyperfine field of the sextet (2) were approximately 30 T after annealing at 700 °C and 25 T after annealing at 800–900 °C.

This significant decrease of the average hyperfine field value with the increase of the annealing temperature indicates that a substantially larger number of Al atoms were incorporated into the Fe(Al) solid solution formed after annealing at 800–900 °C than at 700 °C. Based on the experimental dependence of the average hyperfine field on the composition of binary iron-rich Fe(Al) disordered solid solution, it was estimated that the percent of solute Al atoms increased from about 15% to about 28% after annealing at 700 °C and 800–900 °C, respectively [48,49].

Furthermore, a significant increase of the relative spectral fraction of the sextet (2) from 36% for the sample annealed at 700 °C to 56% and 83% after annealing at 800 °C and 900 °C, respectively, strongly indicates that the higher the annealing temperature, the more effective formation of the Fe(Al) solid solution.

Thermally induced changes in the magnetic Fe-Al environments were accompanied also by the evolution of paramagnetic components in the CEMS spectra. The quadrupole doublet observed after annealing at 700 °C was partially replaced by a single line after annealing at higher temperatures. As concerns the quadrupole doublet (3), its hyperfine parameters suggest the formation of an Al-rich phase with a composition close to Al_5_Fe_2_ [10,50], in good agreement with the XRD data. The appearance of the single line (4) after annealing at 800 and 900 °C strongly indicates the formation of cubic FeAl phase. The isomer shift of the single line was, however, considerably smaller than that characteristic of the ordered intermetallic FeAl phase with the equiatomic composition [49,50]. This fact suggests a non-stoichiometric ratio of the intermetallic phase, i.e., an excess of iron. The relative fraction of the intermetallic spectral component did not exceed 11%. Traces of paramagnetic iron oxides cannot be excluded.

The Mössbauer spectroscopy measurements showed that annealing induced further mixing of iron and aluminum in the samples so that the coating annealed at the highest temperature applied (900 °C) revealed the most homogenous structure, consisting predominantly of the bcc solid solution with an estimated average composition of Fe_72_Al_28_. The presence of a small amount of the intermetallic non-stoichiometric FeAl phase was also observed.

### 3.5. Hardness Analysis

Microhardness analysis has been conducted on the specimens for the cross-section of the coating/substrate system. The Vickers method was used (load 100 g for 10 s). Hardness analysis was realized by means of the Leitz–Wetzlar microhardness tester (LEICA, Wetzlar, Germany). In order to determine the uncertainty of measurement, t-student distribution was conducted, with a confidence level assumed at 95%. The obtained results were used to create a chart that presents the distribution of hardness.

**Figure 13:**
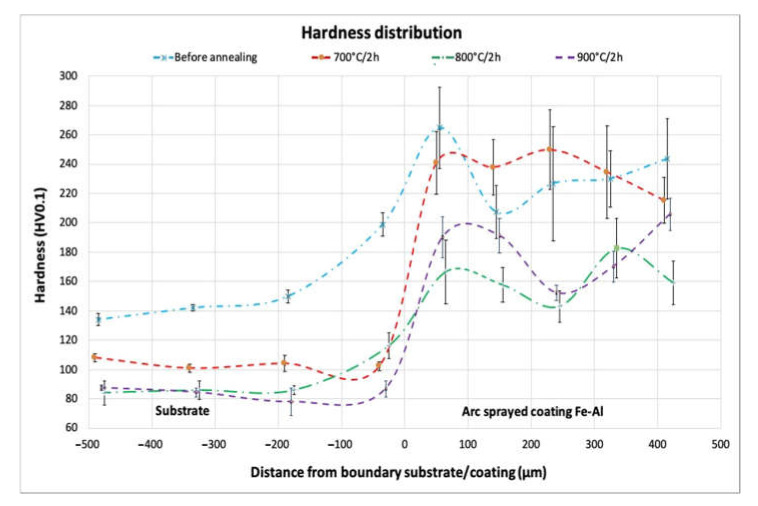
Distribution of microhardness in the cross-section of the substrate material and the arc-sprayed Fe-Al coating, before and after annealing at temperatures 700 °C, 800 °C, and 900 °C.

The microhardness distribution presented in Figure 13 indicates significant differences in the hardness of the arc-spray-deposited coating before annealing and the coatings after the annealing processes, with three different temperature conditions described in heading 2.

Annealing at a temperature of 700 °C did not significantly affect the hardness of the coating; it was comparable with the state before annealing. In addition, a hardness decrease in the steel substrate of approximately 40 HV0.1 was observed for all annealing treatments carried out. Annealing at a temperature of 800 °C caused a noticeable decrease in hardness in the coating by about 80 HV, as an additional effect a reduction in the standard deviation value was observed compared to the untreated coating and the coating annealed at 700 °C. The hardness of the annealed coating at 900 °C was between the hardness of the annealed coatings at 700 °C and 800 °C. In addition, a significant decrease in the value of the standard deviation from the average was noted (for 900 °C it was the lowest among the examined cases, Table 4), which indicates changes in the structure towards homogeneity.

## 4. Conclusions

The results of the SEM/EDX, XRD, CEMS, and HV0.1 experiments and analyses allowed for the evaluation of the changes in a chemical/phase composition and the degree of hardening in the arc-sprayed Fe-Al type coating after annealing at 700 °C, 800 °C, and 900 °C for 2 h.

It was determined that:

- The composite arc-sprayed Fe-Al coating with initial low participation of in situ created intermetallic phases showed significant changes in the phase composition, with an increase in the volume fraction of Fe-Al intermetallic phases as a result of annealing;
- At the lower annealing temperature of 700 °C, besides the Fe(Al) solid solution, other transitional Al-rich Fe2Al5 intermetallic phases were formed;
- An increase in the heating temperature induced further diffusion of Al and the formation of different Fe-Al-type phases, namely, bcc Fe(Al) solid solution and disordered intermetallic FexAly phases with varying chemical compositions (according to the Fe-Al equilibrium system);
- The thermal activation at 800 °C and 900 °C for 2 h stimulated the formation of the FeAl intermetallic phase, with specific {100} reflection originating from a superlattice with B2 ordering (as confirmed in XRD investigations);
- A significant decrease of the bcc Fe metallic phase (from the range of 50% to about 5%) in the arc-sprayed Fe-Al coating was observed, with the increase of annealing temperature up to 900 °C/2 h;
- The volume fraction of the B2 ordered FeAl phase increased with increasing annealing temperature;
- After annealing at the temperature of 900 °C, the structure was composed of a B2 ordered FeAl intermetallic phase and disordered Fe_3_Al secondary solution, confirmed in the Mössbauer spectroscopy investigation;
- Heating of the arc-sprayed Fe-Al coating at a temperature of 900 °C for 2 h initiated the geometrical changes of lamellar structure, which ensured more homogeneity but was still not quite uniform in the SEM/EDX analysis;
- The microhardness distribution indicated significant differences in the hardness of the coatings after the annealing processes with three different temperature conditions;
- Annealing at temperatures of 800 °C and 900 °C caused a noticeable decrease in hardness;
- An additional effect was the reduction of the value of the standard deviation of the mean hardness value with the increase of the annealing temperature. The highest decrease in the value of the standard deviation from the mean hardness value occurred after annealing at the temperature of 900 °C, which confirmed the homogeneity changes of the structure.

## Figures and Tables

**Table 4:** Hardness distribution in cross-section of substrate coating system, before and after annealing; the table shows the average value and standard deviation for the hardness distributions for the charts from Figure 12.

**State** **of the Coating**	**Hardness Distribution, HV0.1**								
	**Approximately Distance from Boundary Substrate/Coating, μm**								
	**−470**	**−325**	**−180**	**−35**	**55**	**150**	**240**	**330**	**420**
Before annealing	134	142	149	198	264.75	207.25	226.75	230	243.5
	(7.91)	(4.3)	(8.32)	(15.99)	(55.36)	(36.31)	(77.82)	(38.22)	(54.55)
700 °C	108	100.83	104.05	102.3	240.75	237.75	249.75	234.5	215.25
	(5.87)	(5.7)	(10.73)	(5.81)	(42.7)	(37.66)	(54.5)	(63.37)	(31.59)
800 °C	84.03	85.75	85.85	116.4	166.5	157.5	143	182.5	159
	(16.46)	(12.53)	(5.85)	(17.82)	(43.87)	(23.69)	(21.44)	(40.6)	(29.67)
900 °C	87.35	84.33	77.83	86.68	190	191.25	152.25	170	205
	(4.12)	(6.24)	(18.38)	(11.11)	(27.61)	(23.59)	(10.13)	(20.99)	(21.83)

## Data Availability

Not applicable.
